# Effects of Sphingosine-1-Phosphate on Cell Viability, Differentiation, and Gene Expression of Adipocytes

**DOI:** 10.3390/ijms21239284

**Published:** 2020-12-05

**Authors:** Xiyuan Wu, Meena Kishore Sakharkar, Martin Wabitsch, Jian Yang

**Affiliations:** 1 Drug Discovery and Development Research Group, College of Pharmacy and Nutrition, University of Saskatchewan, 107 Wiggins Road, Saskatoon, SK S7N 5E5, Canada; xiw178@mail.usask.ca (X.W.); meena.sakharkar@usask.ca (M.K.S.); 2 Department of Pediatrics and Adolescent Medicine, Ulm University Medical Center, Eythstr. 24, 89075 Ulm, Germany; martin.wabitsch@uniklinik-ulm.de

**Keywords:** sphingosine-1-phosphate, preadipocyte, cell viability, adipocyte differentiation, gene expression, transcriptomics

## Abstract

Sphingosine-1-phosphate (S1P) is a highly potent sphingolipid metabolite, which controls numerous physiological and pathological process via its extracellular and intracellular functions. The breast is mainly composed of epithelial cells (mammary gland) and adipocytes (stroma). Adipocytes play an important role in regulating the normal functions of the breast. Compared to the vast amount studies on breast epithelial cells, the functions of S1P in breast adipocytes are much less known. Thus, in the current study, we used human preadipocyte cell lines SGBS and mouse preadipocyte cell line 3T3-L1 as in vitro models to evaluate the effects of S1P on cell viability, differentiation, and gene expression in adipocytes. Our results showed that S1P increased cell viability in SGBS and 3T3-L1 preadipocytes but moderately reduced cell viability in differentiated SGBS and 3T3-L1 adipocytes. S1P was also shown to inhibit adipogenic differentiation of SGBS and 3T3-L1 at concentration higher than 1000 nM. Transcriptome analyses showed that S1P was more influential on gene expression in differentiated adipocytes. Furthermore, our network analysis in mature adipocytes showed that the upregulated DEGs (differentially expressed genes) were related to regulation of lipolysis, PPAR (peroxisome proliferator-activated receptor) signaling, alcoholism, and toll-like receptor signaling, whereas the downregulated DEGs were overrepresented in cytokine-cytokine receptor interaction, focal adhesion, starch and sucrose metabolism, and nuclear receptors pathways. Together previous studies on the functions of S1P in breast epithelial cells, the current study implicated that S1P may play a critical role in modulating the bidirectional regulation of adipocyte-extracellular matrix-epithelial cell axis and maintaining the normal physiological functions of the breast.

## 1. Introduction

Sphingosine-1-phosphate (S1P) is a highly potent sphingolipid metabolite which tightly controls various physiological and pathological processes via its extracellular and intracellular functions [1,2]. Its extracellular function follows an “inside-out” signaling model [3]. S1P is first synthesized from sphingosine by sphingosine kinases 1 (SphK1), which is located in the cytosol. Then, it is transported out of the cell to interact with a family of five G protein-coupled receptors (S1PR_1–5_) to regulate various cellular functions such as proliferation, apoptosis, differentiation, growth, migration, invasion, and angiogenesis [4,5,6,7]. However, the intracellular function of S1P is less understood. It is synthesized by sphingosine kinase 2 (SphK2) either inside the nucleus or in the perinuclear region. S1P then epigenetically affects gene expression by inhibiting histone deacetylase HDAC1 and HDAC2 [8,9,10] and can induce cell apoptosis [11,12].

The breast is a highly specialized female exocrine organ responsible for producing milk to feed offspring. It is structurally divided into ductal epithelium and connective tissue stroma [13]. The ductal epithelium contains luminal, myoepithelial and basal cells and forms the mammary gland [14]. The stroma, which mainly contains adipocytes, plays an important role in regulating morphogenesis, development and homeostasis of the mammary gland [13,15,16]. S1P has been extensively studied for its regulatory role in normal physiology and pathogenesis of the mammary gland [17,18,19,20]. However, its function in breast adipocytes is far from being understood. Limited previous studies showed that S1P inhibits the differentiation of mouse preadipocyte 3T3-L1 cells at concentration higher than 0.5 µM [21]; and modulates adipocyte hypertrophy [22,23,24] and proinflammation response [25].

In addition, adipocytes constitute an abundant source of extracellular matrix (ECM) components in the breast [13,26], which play important roles in cell communication, adhesion, and homeostasis [27,28,29]. S1P can remodel the dynamic ECM and facilitate cell communication between adipocytes and ductal epithelial cells by altering the tight junction assembly [30]. A recent study showed that the level of S1P was about 10 times higher in the interstitial fluid (layer of fluid surrounding the mammary gland) than the mammary gland itself [31]. This implicates that S1P plays a critical role in regulating mammary ductal epithelium, stromal adipocyte tissue and their mutual communications.

To get a better understanding of its functions in adipocytes, we evaluated the effects of S1P on cell viability, differentiation, and gene expression in both human pre- and differentiated adipocyte SGBS cells and mouse pre- and differentiated adipocyte 3T3-L1 cells. It was observed that S1P moderately increased cell viability in preadipocytes but decreased cell viability in differentiated adipocytes, and inhibited adipogenic differentiation at concentration higher than 1 µM. Compared to preadipocytes, the number of gene regulated by S1P was significantly increased in the differentiated adipocytes.

## 2. Results

### 2.1. Effect of S1P on Cell Viability of Pre- and Differentiated Adipocytes

**Figure 1:**
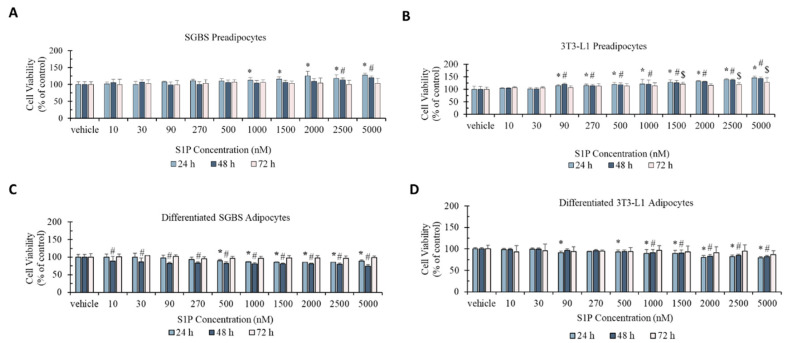
Cell viability (% of control) of preadipocyte SGBS cells (**A**), preadipocyte 3T3-L1 cells (**B**), differentiated adipocyte SGBS cells (**C**) and differentiated adipocyte 3T3-L1 cells (**D**) under sphingosine-1-phosphate (S1P) treatment (10–5000 nm) for 24 h, 48 h, and 72 h, respectively. Cells treated with methanol were used as the vehicle control. Results were shown in mean ± SD and analyzed by one-way ANOVA followed by Dunnett’s post hoc test. Data were calculated from six independent experiments. A significant difference (*) was defined by *p* < 0.05 as compared to control under different time series (* 24 h, ^#^ 48 h, ^$^ 72 h).

The effect of S1P on cell viability was evaluated against pre- and differentiated SGBS and 3T3-L1 adipocytes using the MTT assay (Figure 1). For SGBS preadipocytes, cell viability was statistically significantly increased from 12% at 1000 nM to 28% at 5000 nM after 24 h of S1P treatment and by 10% (*p* < 0.05) at 2500 nM and 20% at 5000 nM (*p* < 0.05) after 48 h of S1P treatment. No statistically significant change in cell viability was observed for any S1P concentration at 72 h of treatment. For 3T3-L1 preadipocytes, cell viability was statistically significantly increased from 15% at 90 nM to 46% at 5000 nM at 24 h of S1P treatment, from 20% at 90 nM to 43% at 5000 nM at 48 h of S1P treatment, and by 21% at 1000 nM, 20% at 2500 nM and 27% at 5000 nM after 72 h of S1P treatment. In general, the effect of S1P on cell viability was stronger in 3T3-L1 preadipocytes and prolonged exposure to S1P weakened the effect.

Cell viability was moderately decreased for both differentiated SGBS and 3T3-L1 adipocytes upon S1P treatment. For differentiated SGBS adipocytes, cell viability was statistically significantly decreased by 10–15% in the concentration of 500–5000 nM after 24 h of S1P treatment, and from 12% at 10 nM to 25% at 5000 nM at 48 h of S1P treatment. For differentiated 3T3-L1 adipocytes, cell viability was statistically significantly decreased from 8% at 90 nM to 21% at 5000 nM (except at 270 nM, *p* > 0.05) at 24 h of S1P treatment and 9% at 1000 nM to 18% at 5000 nM after 48 h of S1P treatment. No statistically significant change in cell viability was observed for both differentiated SGBS and 3T3-L1 adipocytes for 72 h of S1P treatment at any concentration. Similar to preadipocytes, prolonged treatment weakened the effect of S1P in differentiated adipocytes.

The opposite effects on cell viability suggested that S1P elicited different functions between pre- and differentiated adipocytes.

### 2.2. Effect of S1P on Adipocyte Differentiation

**Figure 2:**
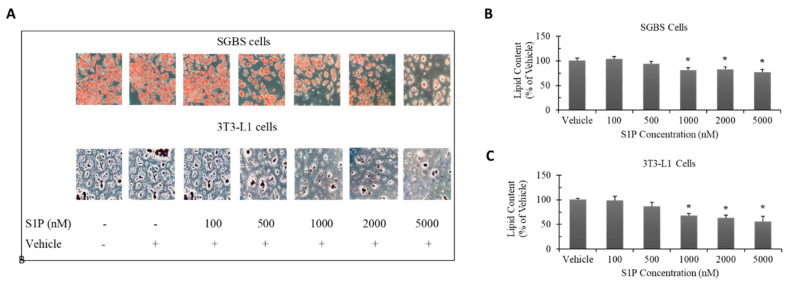
Changes in lipid accumulation (% of vehicle) in differentiated SGBS and 3T3-L1 adipocytes with S1P treatment throughout the whole differentiation process (14 days for SGBS and 10 days for 3T3-L1). SGBS and 3T3-L1 preadipocytes were induced to differentiate under S1P treatment (concentration: 100–5000 nM). Oil red O staining was applied to visualize lipid droplets accumulated inside differentiated adipocytes. Photos were taken under a light microscope at 200× magnification (**A**). The staining solution was extracted and quantified by absorbance of 492 nm, which represents the lipid content inside the differentiated SGBS adipocytes (**B**) and 3T3-L1 adipocytes (**C**). The results were represented as mean ± SD and analyzed by one-way ANOVA followed by Dunnett’s post hoc comparisons. Data were calculated from three independent experiments. A significant difference (*) was defined by *p* < 0.05 as compared with vehicle control.

To examine the effect of S1P on adipocyte differentiation, we treated the SGBS and 3T3-L1 preadipocytes with S1P (concentrations: 100, 500, 1000, 2000 and 5000 nM) throughout the whole differentiation process (14 days for SGBS and 10 days for 3T3-L1). The lipid content was much higher in differentiated SGBS adipocytes than differentiated 3T3-L1 adipocytes (Figure 2A). Furthermore, statistically significant decrease in lipid content was observed in both differentiated adipocytes when S1P concentration was higher than 1000 nM (Figure 2B,C). For differentiated SGBS adipocytes, the lipid content was reduced by 19% at 1000 nM, 19% at 2000 nM and 23% at 5000 nM; whereas for differentiated 3T3-L1 adipocytes, the lipid content was reduced by 32% at 1000 nM, 37% at 2000 nM and 44% at 5000 nM. Apparently, S1P exerted a much stronger inhibitory effect on differentiation in 3T3-L1 cells. Our current observation is consistent with previous studies [21].

### 2.3. Effect of S1P on Gene Expression in Pre- and Differentiated Adipocytes

**Figure 3:**
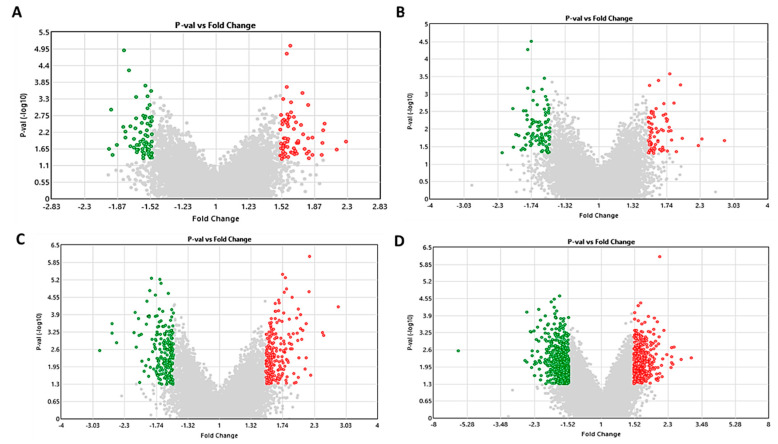
Volcano plots [fold change vs. *p*-value (℡log_10_)] displaying differentially expressed genes (DEGs) in pre- and differentiated adipocytes treated with 100 nM S1P compared to vehicle control. Genes with fold change ≤ −1.5 (green) and ≥1.5 (red) and *p* < 0.05 are highlighted in SGBS preadipocyte (**A**), 3T3-L1 preadipocyte (**B**), differentiated SGBS adipocyte (**C**) and differentiated 3T3-L1 adipocyte (**D**).

The gene expression affected by S1P treatment (concentration: 100 nM) was investigated by transcriptomics using the Affymetrix Clariom™ S Mouse/Human Array (Thermo Fisher Scientific). Differentially expressed genes (DEGs) were highlighted in green (fold change ≤ −1.5 and *p* < 0.05) and red (fold change ≥ 1.5 and *p* < 0.05) in the volcano plots (Figure 3). We identified 139 DEGs in SGBS preadipocytes, 178 DEGs in 3T3-L1 preadipocytes, 411 DEGs in differentiated SGBS adipocytes and 974 DEGs in differentiated 3T3-L1 adipocytes, respectively. The top 30 up- and downregulated DEGs are summarized in Table 1.

### 2.4. Gene Ontology (GO) Analysis

**Figure 4:**
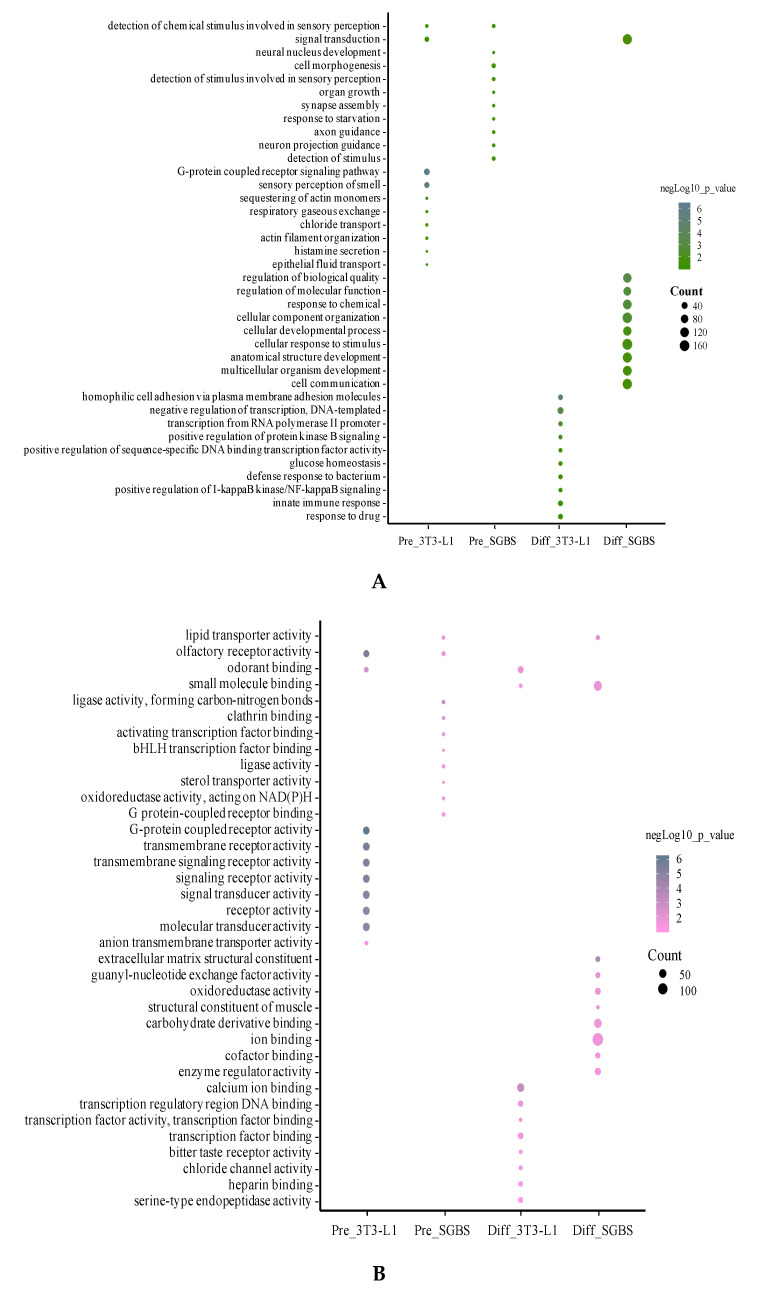

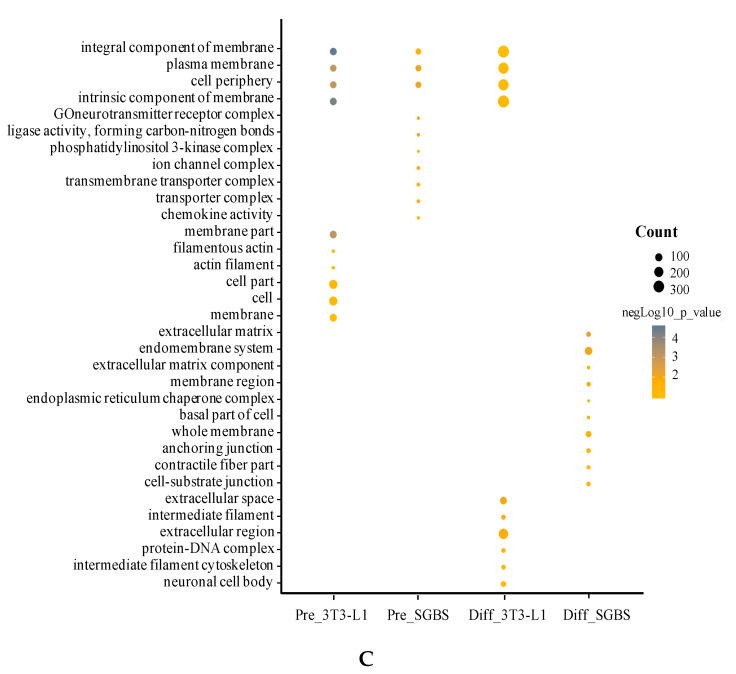
Top 10 enriched GO terms in biological process (**A**), molecular function (**B**), and cellular component (**C**). For each cell type, the DEG list was subjected to GO analysis and the most enriched GO terms were summed up. Size of the bubbles indicates counts of genes identified in each GO term, and colors of the bubbles show significance (−log_10_ *p*-value) of GO enriched fold changes compared to control.

We analyzed the GO terms for DEGs in pre- and differentiated SGBS and 3T3-L1 adipocytes using ConsensusPathDB [32]. The top 10 enriched GO terms (*p* < 0.05) in biological process, molecular function and cellular component are summarized in Figure 4. It was obvious that the enriched GO terms were different between pre- and differentiated adipocytes and between SGBS and 3T3-L1.

### 2.5. Pathway Entichment Analysis

**Figure 5:**
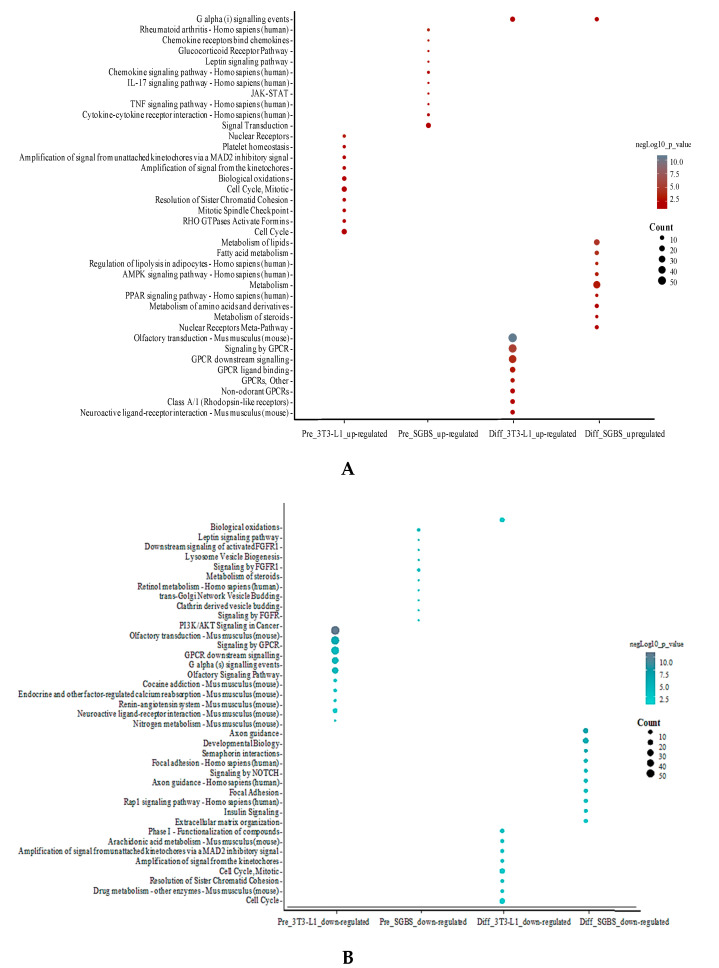
Pathway enrichment analysis in pre- and differentiated SGBS and 3T3-L1 adipocytes. The top 10 enriched pathways for upregulated DEGs (**A**) and downregulated DEGs (**B**) DEGs are plotted separately. Size of the bubbles indicated the count of genes identified in each pathway, and color of the bubbles show significance (−log_10_ *p*-value) of enriched pathway compared to control.

Up- and downregulated DEGs (|fold change| ≥ 1.5 and *p* < 0.05) were individually subjected to pathway enrichment using ConsensusPathDB. For the upregulated DEGs, the enriched pathways were inflammatory signaling and cytokine involved pathways in SGBS preadipocytes; cell cycle/mitosis related pathways in 3T3-L1 preadipocytes; lipid metabolism-related pathways (e.g., PPAR signaling pathway, metabolism of fatty acid, metabolism, and metabolism of lipids) in differentiated SGBS adipocytes; and G protein-coupled receptor signaling-related pathways (e.g., signaling by GPCR, GPCR ligand binding, and GPCR downstream signaling) in differentiated 3T3-L1 adipocytes, respectively (Figure 5A). For the downregulated DEGs, the enriched pathways were identified to be metabolite regulation associated pathways in SGBS preadipocytes; GPCR signaling and olfactory transduction pathways in 3T3-L1 preadipocytes; focal adhesion and axon guidance related pathways in differentiated SGBS adipocytes; and cell cycle and cell mitosis-related pathways (e.g., cell cycle, mitotic and amplification of signal from the kinetochores) in differentiated 3T3-L1 adipocytes, respectively (Figure 5B).

## 3. Discussion

**Figure 6:**
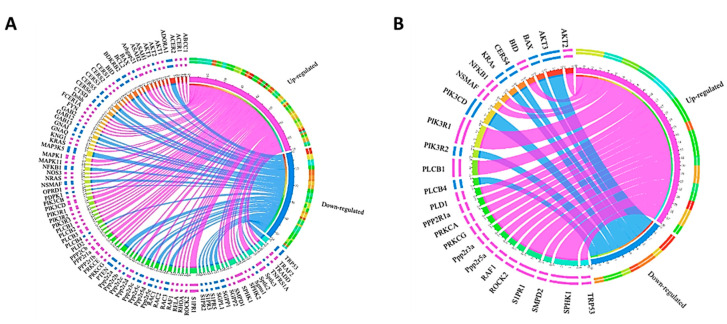
Chord diagrams showing genes with significantly different expressions (*p* < 0.05) between differentiated and undifferentiated SGBS cells (**A**) and 3T3-L1 cells (**B**) upon 100 nM S1P treatment. The up- and downregulated genes are shown in magenta and blue, respectively.

In the current study, we show the opposite effects of S1P on cell viability of adipocytes. S1P enhanced cell viability in human SGBS and mouse 3T3-L1 preadipocytes, but moderately decreased cell viability in differentiated SGBS and 3T3-L1 adipocytes. S1P also inhibited lipid accumulation during differentiation in both adipocytes at concentration higher than 1000 nM. Our observation was in line with previous studies that S1P enhanced cell proliferation and suppressed adipogenesis of preadipocytes [21,33,34]. However, these previous studies were mainly carried out with mouse preadipocytes (e.g., 3T3-L1 and 3T3-F442A). The inhibition of adipogenesis was evaluated by the downregulation of adipocyte-specific transcriptional factors [34]. A high concentration of S1P was shown to activate cAMP/PKA (cyclic AMP/protein kinase A), possibly via S1PR_1/2_, to promote lipolysis and inhibit lipid accumulation [35]. However, the expressions of genes *PRKACA* and *PRKACB* were not changed by S1P treatment in pre- and differentiated SGBS adipocytes. Similarly, the expressions of mouse orthologs *Prkaca* and *Prkacb* were not altered by S1P stimulus in pre- and differentiated 3T3-L1 adipocytes. To explain the different effects of S1P between pre- and differentiated adipocytes, we identified genes with significantly different expression (*p* < 0.05) between differentiated and undifferentiated (i.e., pre-) SGBS and 3T3-L1 adipocytes. As shown in Figure 6, the differentially expressed genes were mainly involved in the PI3K-AKT pathway (e.g., *AKT1-3* and *PI3KR1-3*), sphingolipid metabolism and S1P signaling pathway (e.g., *CERS1-6*, *SPHK1-2* and *S1PR1-5*) and other protein kinase/phosphatase signaling (e.g., *ROCK2*, *PLCB1-4* and *PPP2Rs*). However, these genes were regulated differently between SGBS and 3T3-L1 adipocytes. Specifically, genes *SPHK1* and *S1PR1* were upregulated in both differentiated SGBS and 3T3-L1 adipocytes compared to their respective preadipocytes, implicating that the SphK1-S1P-S1PR_1_ signaling axis was enhanced. Because this signaling axis normally promotes cell proliferation, other S1P-mediated signaling pathways must be involved in decreasing cell viability in the differentiated SGBS and 3T3-L1 adipocytes. Moreover, the expressions of *S1PR2*, *S1PR3* and *S1PR5* were downregulated in the differentiated SGBS adipocytes, suggesting that S1P-S1PR_2_, S1P-S1PR_3_ and S1P-S1PR_5_ signaling pathways were likely to be impaired. Further studies are warranted to identify which signaling pathway is responsible for the reduced proliferation of differentiated SGBS and 3T3-L1 adipocytes.

Out of the DEGs identified from transcriptomics study, 9% and 17% were involved in human secretome in pre- and differentiated SGBS adipocytes, respectively. Furthermore, about 2% of the DEGs were in the adipokine secretome in the differentiated SGBS adipocytes. This implicated that S1P may play an important role in regulating the adipocyte secretory pattern, which, in turn, controls the normal functions of adipocytes and their communications with other types of cells. Taking into consideration that the breast is mainly composed of epithelial cells and adipocytes and S1P is accumulated to high concentration in the interstitial fluid [13,14,31], the current study suggests that S1P is highly likely to be crucial for the normal functions of both epithelial cells and adipocytes, as well as their mutual communications, in the breast.

**Figure 7:**
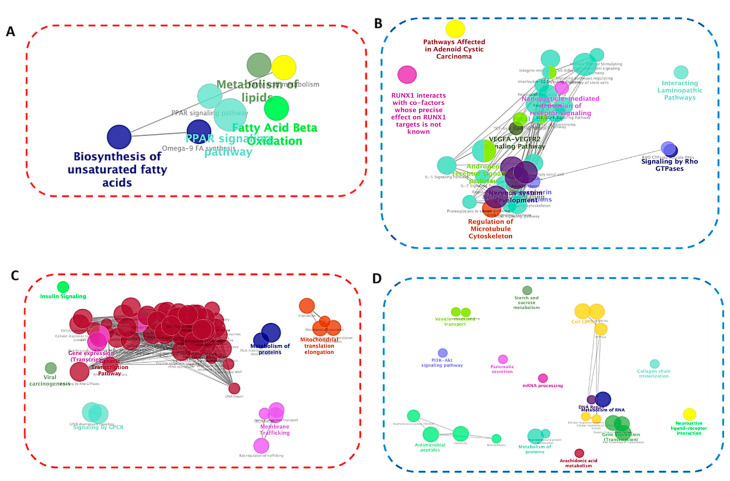
Pathway network analysis based on up- and downregulated DEGs in differentiated SGBS (**A** and **B**, respectively) and 3T3-L1 (**C** and **D**, respectively) adipocytes. The analysis was performed using ClueGO. Enriched pathways were obtained from the KEGG and WikiPathway databases and grouped based on shared genes (ranging from red circle: upregulated to blue circle: downregulated). Size of the nodes indicates degree of significance, where only the most significant term/pathway is labeled as the name of the group. Function-related networks are grouped and may partially overlap.

Network analysis was performed using Cytoscape [36] to investigate the biological relevance of the identified pathways on lipid metabolism in mature adipocytes. Multiple functions of the upregulated DEGs were related to regulation of lipolysis, PPAR signaling, alcoholism, and toll-like receptor signaling (Figure 7A,C). The downregulated DEGs were overrepresented in cytokine–cytokine receptor interaction, focal adhesion, starch and sucrose metabolism, and nuclear receptors pathways (Figure 7B,D). Thus, the functional role of S1P in lipid metabolism involved regulation of cytokines, receptors signaling and focal adhesion. It is noteworthy that PPARs are ligand-activated transcription factors. The PPAR pathway, which is activated by fatty acids and their derivatives regulates numerous biological processes, such as adipocyte differentiation, lipid/glucose metabolism, and energy homeostasis.

**Figure 8:**
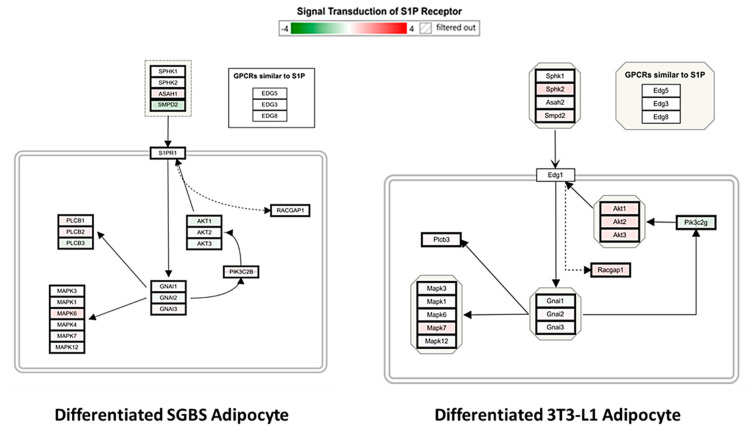
Mapping the S1P-S1PR_1_ signaling pathway in differentiated SGBS and 3T3-L1 adipocytes. WikiPathway was used to identify and visualize genes involved in the S1P-S1PR_1_ signaling axis in S1P-treated differentiated SGBS and 3T3-L1 adipocytes compared to the vehicle control. S1PR_1_ refers to sphingosine-1-phosphate receptor 1.

We further mapped genes of the S1P-S1PR_1_ signaling pathway in the S1P-treated differentiated SGBS and 3T3-L1 adipocytes. As shown in Figure 8, the regulation of S1P-S1PR_1_ signaling axis was significantly different between SGBS and 3T3-L1 cells. A unique feature is that genes *AKT1, AKT2 and AKT3* were downregulated in differentiated SGBS adipocytes, whereas the ortholog genes *Akt1, Akt2 and Akt3* were upregulated in differentiated 3T3-L1 adipocytes. AKTs are downstream common targets of multiple signaling pathways other than S1P1-S1PR_1_, such as epidermal growth factor receptor (EGFR) signaling pathway and insulin receptor (IR) signaling pathway, and play important roles in cell proliferation, migration and survival [37,38,39]. Thus, the current results implicated that regulation of S1P-S1PR_1_ and crosstalk between S1P-S1PR_1_ and other signaling pathways in mature adipocytes might be species-specific. Further studies are required to identify the exact differences of S1P treatment between mouse and human adipocytes and investigate the transferability of mouse-based study results to human health research.

## 4. Materials and Methods

### 4.1. Materials

Sphingosine-1-phosphate (S1P), biotin, d-pantothenic acid hemicalcium salt, apo-transferrin (human), insulin, 3,3′,5-triiodo-l-thyronine sodium salt (T3), rosiglitazone, dexamethasone, 3-isobutyl-1-methylxanthine (IBMX), hydrocortisone, fetal bovine serum (FBS), Oil red O, and 3-(4,5-dimethylthiazol-2-yl)-2,5-diphenyltetrazolium bromide (MTT) were purchased from Sigma-Aldrich Canada (Oakville, ON, Canada). Trypsin-EDTA (0.25%), phosphate-buffered saline (PBS) and penicillin-streptomycin were purchased from HyClone Laboratories Inc. (Logan, UT, USA). Triton X-100 (98%) and Dulbecco’s phosphate-buffered saline (DPBS) was purchased from Thermo Fisher Scientific (Burlington, ON, Canada). Human preadipocyte cell line SGBS, which was derived from an infant with Simpson–Golabi–Behmel syndrome, was established in coauthor Martin Wabitsch’s laboratory. Mouse preadipocyte cell line 3T3-L1 was kindly provided by Professor Jane Alcon, College of Pharmacy and Nutrition, University of Saskatchewan, Canada. Dulbecco’s modified Eagle’s medium (DMEM) and 1:1 mixture of Dulbecco’s modified eagle’s medium with ham’s F12 medium (DMEM-F12) were purchased from Gibco (Thermo Fisher Scientific, Burlington, ON, Canada).

### 4.2. Cell Culture of Preadipocytes

Both cell lines were cultured under preadipocyte expansion media in T-75 cell culture flasks at 37 °C with a humidified atmosphere of 5% CO_2_. Human preadipocyte cell line SGBS was cultured in DMEM-F12 supplemented with 10% FBS, 1% penicillin/streptomycin, and 10% 5 mM pantothenate/biotin mixture (Pan/Bio; pantothenate:biotin = 1.7:3.3). Mouse preadipocyte cell line 3T3-L1 was cultured in DMEM containing 10% FBS. The preadipocyte expansion media were changed every two to three days.

### 4.3. Adipocyte Differentiation

After reaching 80–90% confluency (day 0), SGBS preadipocytes were washed with DPBS and then induced to differentiate in serum-free differentiation media (DMEM-F12 supplemented with 1% penicillin/streptomycin, 10% 5 mM Pan/Bio, 0.01 mg/mL human transferrin, 100 nM hydrocortisone, 0.2 nM T3, 25 nM dexamethasone, 250 µM IBMX, 2 µM rosiglitazone and 20 nM insulin). Four days after induction (day 4), the differentiation media were changed to postdifferentiation media by excluding IBMX, dexamethasone and rosiglitazone. The cells were cultured for 10 more days with media changed every four days. The SGBS cells were fully differentiated in about 8–12 days after induction as evidenced by changes in internal lipid droplet accumulation [40].

To differentiate 3T3-L1 preadipocytes, the cells were first cultured in preadipocyte expansion media for two days after reaching full confluence and before inducing differentiation (day 0). The cells were washed with DPBS and then induced to differentiate in differentiation media (DMEM containing 10% FBS, 1.0 µM dexamethasone, 0.5 mM IBMX and 1.0 µg/mL insulin) for another two days. On day 2, the media were replaced with the postdifferentiation media (DMEM with 10% FBS and 1.0 µg/mL insulin) and cultured for another six days. Cell culture media were changed every two to three days. Differentiation of 3T3-L1 cells were also evidenced by changes in internal lipid droplet accumulation.

### 4.4. Cell Viability Assay (MTT Assay)

The effect of S1P on cell viability of pre- and differentiated SGBS and 3T3-L1 adipocytes were measured using the colorimetric MTT assay [41]. Briefly, for each adipocyte cell line, preadipocytes were seeded at 8 × 10^3^ cells/well and differentiated adipocytes were seeded at 1 × 10^4^ cells/well in 96 well plates. The seeding numbers were selected based on a pilot study. The cells were allowed to attach for 24 h before being treated with S1P (concentration: 10–5000 nM) for 24 h, 48 h, and 72 h, respectively. Methanol was used as a vehicle control. At the end of S1P treatment, the media were replaced with 100 µL MTT (0.5 mg/mL) dissolved in corresponding complete media and incubated for 4 h. Then, the media containing MTT were replaced with 150 µL DMSO to dissolve the formed formazans under constant shaking for 15 min on an orbital shaker. Absorbance was subsequently measured at 570 nm using a BioTek Synergy HK microplate reader (BioTek, Winooski, VT, USA). Cell viability was interpreted as the absorption ratio of treatment to control. The experiment was carried out in triplicate with six replicates in each test, and the data were shown in mean value ± SD as analyzed by GraphPad Prism 8 software (San Diego, CA, USA).

### 4.5. Oil Red O Staining

Oil red O staining was used to measure lipid accumulation in differentiated SGBS and 3T3-L1 adipocytes. SGBS or 3T3-L1 preadipocytes were seeded at 2 × 10^4^ cells/well in 24-well plates and cultured with preadipocyte complete media before differentiation induction. On the induction day (day 0), media were replaced with differentiation media +/− S1P (concentration: 100–5000 nM). After induction, the media were changed to postdifferentiation media +/− S1P for SGBS on day 4 or 3T3-L1 on day 2. Media or media + S1P were changed every four days for SGBS or 2–3 days for 3T3-L1 throughout the whole adipogenic differentiation phase. On day 14 for SGBS or day 8 for 3T3-L1, lipid accumulation in differentiated adipocytes was measured using Oil red O staining. Briefly, cells were first washed with 1× PBS twice after removal of media and then fixed with 4% paraformaldehyde in 1× PBS at room temperature for 30 min. The cells were stained with 0.2% Oil red O working solution for 15 min after washing with 1× PBS twice. Stained cells were washed with ddH2O until there was no pink color observed. Images were captured using an Olympus inverted phase microscope (Olympus Canada, Richmond Hill, ON, Canada) at 200× magnification after wells were dried. Then, 2-propanol was used to solubilize the Oil red O dye, and the extraction was seeded in 96-well plates for lipid quantification by measuring absorbance at 492 nm using a BioTek Synergy HK microplate reader. Differentiation media + methanol was used as a vehicle control. Preadipocytes stained with Oil red O solution was used as a blank control. The results were presented as absorbance percentage normalized to control (100%) using the following formula:

(A_treat_ − A_blank_)/(A_control_ − A_blank_) × 100% (1)

### 4.6. Transcriptome Analysis

Pre- and differentiated SGBS and 3T3-L1 and SGBS adipocytes were treated with 100 nM S1P for 24 h before being collected by centrifugation. Treatment with methanol was used as a vehicle control. The collated cell pellets were sent to Applied Biosystems Microarray Research Services Laboratory (Thermo Fisher Scientific, Burlington, ON, Canada) for RNA extraction and transcriptome analysis. Extracted RNA samples were analyzed using the Affymetrix Clariom™ S Mouse/Human Array, which covers all known well-annotated genes. Raw data were normalized by Robust Multiple Averaging algorithms.

### 4.7. DEG Analysis

For each adipocyte transcriptome analysis, gene expression values of the S1P-treated group were compared to methanol control group. Differentially expressed genes (DEGs) were defined as genes with |fold change| ≥ 1.5 and *p* < 0.05 controlled by a false discovery rate (FDR). DEGs of the differentiated SGBS adipocytes were filtered for secreted protein-coding genes based on the human secretome list from the Human Protein Atlas database [42], including those identified as proteins released from adipocytes [43].

### 4.8. Gene Ontology (GO) and Pathway Enrichment Analyses

GO and pathway enrichment analyses were performed on DEGs (|fold change| ≥ 1.5, *p* < 0.05, FDR controlled) identified from pre- and differentiated SGBS and 3T3-L1 adipocytes. Overrepresentation analysis (ORA) was performed using Consensus PathDB [32]. Each DEG list was input for defining overrepresented sets in two categories: GO-based sets and pathway-based sets. GO annotations were categorized into three GO terms: biological processes, molecular functions, and cellular components. Significant enrichment was calculated using hypergeometric testing with Benjamini–Hochberg FDR correction (cut-off of FDR < 0.05). Pathway enrichment analysis was only performed on DEGs from differentiated SGBS and 3T3-L1 adipocytes by employing the ORA tool in Consensus PathDB. Up- and downregulated DEGs were analyzed separately. We searched for pathways from three databases, KEGG [44,45], Reactome [46] and Wikipathway [47]. Minimal overlap with the input list of 2 and *p*-value cut-off at 0.05 were set as the default.

### 4.9. Statistical Analysis

GraphPad Prism 8 was used to perform data analysis. Data for the MTT assay were calculated from three independent experiments with each experiment containing six replicates. Data for other analyses were calculated from three independent experiments with each experiment containing three replicates. Results were presented as mean ± SD. Data comparison was performed using the analysis of variance (ANOVA, one-way or two-way), followed by Dunnett’s or Tukey’s post hoc tests. Results were considered statistically significant for *p* < 0.05.

## 5. Conclusions

The breast, which is mainly composed of epithelial cells and adipocytes, is a highly specialized female exocrine organ responsible for producing milk to feed offspring. Because S1P can accumulate up to 10 times higher in the interstitial fluid than the mammary gland itself, it may be essential for extracellular matrix remodeling and communications between epithelial cells and adipocytes. In this study, we showed that S1P exerted potent effects not only on adipocyte differentiation but also on cell viability and gene expression in both pre- and differentiated adipocytes. S1P-regulated pathways, such as lipolysis, PPAR signaling and cytokine–cytokine receptor interaction, will be investigated in our future studies to illustrate how S1P regulates the adipocyte-extracellular matrix-epithelial cell axis to maintain normal physiological functions of the breast and prevent development of breast cancer.

## Figures and Tables

**Table 1:** The top 30 up- and downregulated DEGs in pre- and differentiated SGBS and 3T3-L1 adipocytes.

Upregulated DEGs				Downregulated DEGs			
*Pre-* *SGBS*	*Diff-* *SGBS*	*Pre-* *3T3-L1*	*Diff-* *3T3-L1*	*Pre-* *SGBS*	*Diff-* *SGBS*	*Pre-* *3T3-L1*	*Diff-* *3T3-L1*
*PIK3C2B*	*HAS1*	*Gm17332*	*Fstl1*	*FGF20*	*EMX2*	*Tmsb15b2*	*Stard9*
*CTPS2*	*CIDEC*	*Ces2c*	*Gm8281*	*OLFM3*	*ACAN*	*Gm14092*	*Krtap16-3*
*ZNF506*	*TNFRSF19*	*Olfr1477*	*Mier2*	*FCER1A*	*IFIT1*	*Gm13298*	*Folr1*
*UBE2D3*	*ZNF141*	*Sftpc*	*Gm2237*	*MRAP*	*SETD3*	*Wdr17*	*Usp26*
*TRABD2A*	*GPRC5A*	*Vmn1r46*	*Atg7*	*PHYHIPL*	*IRAK1*	*Slc25a31*	*Mcoln2*
*OSBPL1A*	*KLHL13*	*Tcp11l1*	*Lacc1*	*CEMIP*	*FYCO1*	*Gm20823*	*Gad2*
*CISH*	*THRSP*	*Pou2f1*	*Depdc7*	*KLHDC8B*	*DENND3*	*Olfr1436*	*Gm17482*
*BIRC3*	*ADAMTS9*	*Ssx9*	*Adipoq*	*CDKAL1*	*NEO1*	*Vmn1r227*	*Gm17428*
*SCRIB*	*CAT*	*Olfr810*	*B3gnt9*	*TMEM170B*	*ARID1A*	*Olfr1323*	*Pygl*
*CCDC54*	*SYN2*	*Gm14025*	*Trim13*	*FOXO1*	*STPG2*	*Olfr825*	*Ubash3b*
*OR1B1*	*CLHC1*	*Gm3127*	*Slx4*	*SHISA4*	*PODN*	*Ccdc152*	*Rnase9*
*PLSCR5*	*LIPE*	*Olfr807*	*Lrrc9*	*DIRC3*	*HPS1*	*Iqcj*	*Fam19a1*
*SPATA5*	*AGBL2*	*Lctl*	*Eif6*	*PDE6A*	*CDH22*	*Afp*	*Chd7*
*SLC25A25*	*HACD3*	*Clec7a*	*Rcor2*	*BRD9*	*TTLL12*	*Oas1a*	*Gm11111*
*OR11H12*	*GK*	*Ifna11*	*Esp36*	*FRY*	*RASSF3*	*Gm20738*	*Vpreb2*
*LOC340074*	*NAT2*	*Atp8a1*	*Arhgap33*	*QRSL1*	*MICAL2*	*Gm10477*	*Gm2745*
*NDUFA8*	*PDE11A*	*Sema3d*	*Ensa*	*FDXR*	*RGS7*	*Olfr809*	*Col16a1*
*KSR1*	*CST2*	*5330417C22Rik*	*Sh2b1*	*C17orf64*	*ATP11A*	*Gm5662*	*Prlr*
*API5L1*	*CD207*	*Olfr392*	*Pcdhb21*	*KIRREL3*	*LCTL*	*Gm19668*	*Gm4399*
*CCDC160*	*SENP8*	*Klra23*	*Sema4g*	*LY6G6C*	*PITPNM2*	*Hsfy2*	*Gm4406*
*OR2F1*	*PDE1B*	*Gm10037*	*D430020J02Rik*	*MUC3A*	*ADRBK1*	*Rhbdl3*	*Arhgef26*
*LONRF1*	*EPHA4*	*Olfr133*	*Saal1*	*DEFB104A*	*MINK1*	*Tmprss11c*	*Pnmal1*
*TRIM49D2*	*BCO1*	*4930402K13Rik*	*Olfr1076*	*C9orf50*	*LTBP2*	*Lrit2*	*Defa3*
*POGZ*	*HPD*	*Pde9a*	*Sssca1*	*TMEM129*	*MAN2A2*	*S100a14*	*Defa17*
*TACC3*	*LAIR1*	*Vmn1r4*	*Fbxw17*	*LAGE3*	*SCUBE3*	*Gm5538*	*Vmn2r110*
*PTK2*	*SLC38A4*	*Fpr-rs6*	*Zadh2*	*SLC2A7*	*GSK3A*	*Mlana*	*Lrrtm3*
*STK32A*	*ADIPOQ*	*3425401B19Rik*	*Abi3bp*	*KRTAP1–5*	*MKL*	*Sult2a2*	*LOC102642717*
*CCL20*	*LIPA*	*Lama3*	*Fam180a*	*RAB25*	*RFESD*	*Olfr773*	*Gm10436*
*GMPR*	*SMOX*	*Pdk2*	*Mtor*	*FLOT1*	*CCDC181*	*Car3*	*Olfr1258*
*C17orf47*	*NME8*	*Catsperd*	*Foxm1*	*MYO1E*	*RGS21*	*Elmod1*	*Defb25*

## References

[1] Cartier A., Hla T. (2019). Sphingosine 1-phosphate: Lipid signaling in pathology and therapy. Science.

[2] Pyne N.J., Adams D.R., Pyne N.J. (2016). Sphingosine 1-phosphate and sphingosine kinases in health and disease: Recent advances. Prog. Lipid Res..

[3] Takabe K., Paugh S.W., Milstien S., Spiegel S. (2008). “Inside-out” signaling of sphingosine-1-phosphate: Therapeutic targets. Pharmacol. Rev..

[4] Cheng J.-C., Wang E.Y., Yi Y., Thakur A., Tsai S.-H., Hoodless P.A. (2018). S1P Stimulates Proliferation by Upregulating CTGF Expression through S1PR2-Mediated YAP Activation. Mol. Cancer Res..

[5] Baeyens A.A., Schwab S.R. (2020). Finding a Way Out: S1P Signaling and Immune Cell Migration. Annu. Rev. Immunol..

[6] Rohrbach T., Maceyka M., Spiegel S. (2017). Sphingosine kinase and sphingosine-1-phosphate in liver pathobiology. Crit. Rev. Biochem. Mol. Biol..

[7] Dai L., Liu Y., Xie L., Wu X., Qiu L., Di W. (2017). Sphingosine kinase 1/sphingosine-1-phosphate (S1P)/S1P receptor axis is involved in ovarian cancer angiogenesis. Oncotarget.

[8] Hait N.C., Allegood J., Maceyka M., Strub G.M., Harikumar K.B., Singh S.K., Luo C., Marmorstein R., Kordula T., Milstien S. (2009). Regulation of Histone Acetylation in the Nucleus by Sphingosine-1-Phosphate. Science.

[9] Maceyka M., Harikumar K.B., Milstien S., Spiegel S. (2012). Sphingosine-1-phosphate signaling and its role in disease. Trends Cell Biol..

[10] Ebenezer D.L., Fu P., Suryadevara V., Zhao Y., Natarajan V. (2016). Epigenetic regulation of pro-inflammatory cytokine secretion by sphingosine 1-phosphate (S1P) in acute lung injury: Role of S1P lyase. Adv. Biol. Regul..

[11] Sankala H.M., Hait N.C., Paugh S.W., Shida D., Elmore L.W., Dent P., Milstien S., Spiegel S., Lepine S. (2007). Involvement of Sphingosine Kinase 2 in p53-Independent Induction of p21 by the Chemotherapeutic Drug Doxorubicin. Cancer Res..

[12] Igarashi N., Okada T., Hayashi S., Fujita T., Jahangeer S., Nakamura S.-I. (2003). Sphingosine Kinase 2 Is a Nuclear Protein and Inhibits DNA Synthesis. J. Biol. Chem..

[13] Hovey R.C., Aimo L. (2010). Diverse and Active Roles for Adipocytes During Mammary Gland Growth and Function. J. Mammary Gland. Biol. Neoplasia.

[14] Cristea S., Polyak K. (2018). Dissecting the mammary gland one cell at a time. Nat. Commun..

[15] Zwick R.K., Rudolph M.C., Shook B.A., Holtrup B., Roth E., Lei V., Van Keymeulen A., Seewaldt V., Kwei S., Wysolmerski J. (2018). Adipocyte hypertrophy and lipid dynamics underlie mammary gland remodeling after lactation. Nat. Commun..

[16] Landskroner-Eiger S., Park J., Israel D., Pollard J.W., Scherer P.E. (2010). Morphogenesis of the developing mammary gland: Stage-dependent impact of adipocytes. Dev. Biol..

[17] Singh S.K., Spiegel S. (2020). Sphingosine-1-phosphate signaling: A novel target for simultaneous adjuvant treatment of triple negative breast cancer and chemotherapy-induced neuropathic pain. Adv. Biol. Regul..

[18] Tsuchida J., Nagahashi M., Takabe K., Wakai T. (2017). Clinical Impact of Sphingosine-1-Phosphate in Breast Cancer. Mediat. Inflamm..

[19] Wang W., Hind T., Lam B.W.S., Herr D.R. (2019). Sphingosine 1–phosphate signaling induces SNAI2 expression to promote cell invasion in breast cancer cells. FASEB J..

[20] Li S., Zhou Y., Zheng X., Wu X., Liang Y., Wang S., Zhang Y. (2016). Sphk1 promotes breast epithelial cell proliferation via NF-κB-p65-mediated cyclin D1 expression. Oncotarget.

[21] Moon M.-H., Jeong J.-K., Lee Y., Seol J.-W., Park S. (2014). Sphingosine-1-phosphate inhibits the adipogenic differentiation of 3T3-L1 preadipocytes. Int. J. Mol. Med..

[22] Anderson A.K., Lambert J.M., Montefusco D.J., Tran B.N., Roddy P., Holland W.L., Cowart L.A. (2020). Depletion of adipocyte sphingosine kinase 1 leads to cell hypertrophy, impaired lipolysis, and nonalcoholic fatty liver disease. J. Lipid Res..

[23] Kitada Y., Kajita K., Taguchi K., Mori I., Yamauchi M., Ikeda T., Kawashima M., Asano M., Kajita T., Ishizuka T. (2016). Blockade of Sphingosine 1-Phosphate Receptor 2 Signaling Attenuates High-Fat Diet-Induced Adipocyte Hypertrophy and Systemic Glucose Intolerance in Mice. Endocrinology.

[24] Moon M.-H., Jeong J.-K., Lee J.-H., Park Y.-G., Lee Y.-J., Seol J.-W., Park S. (2012). Antiobesity activity of a sphingosine 1-phosphate analogue FTY720 observed in adipocytes and obese mouse model. Exp. Mol. Med..

[25] Wang J., Badeanlou L., Bielawski J., Ciaraldi T.P., Samad F. (2014). Sphingosine kinase 1 regulates adipose proinflammatory responses and insulin resistance. Am. J. Physiol. Metab..

[26] Rybinska I., Agresti R., Trapani A., Tagliabue E., Triulzi T. (2020). Adipocytes in Breast Cancer, the Thick and the Thin. Cells.

[27] Boudreau N., Bissell M.J. (1998). Extracellular matrix signaling: Integration of form and function in normal and malignant cells. Curr. Opin. Cell Biol..

[28] Todd J.R., Ryall K.A., Vyse S., Wong J.P., Natrajan R.C., Yuan Y., Tan A.-C., Huang P.H. (2016). Systematic analysis of tumour cell-extracellular matrix adhesion identifies independent prognostic factors in breast cancer. Oncotarget.

[29] Sonbol H.S. (2018). Extracellular matrix remodeling in human disease. J. Microsc. Ultrastruct..

[30] Hung R.-J., Hsu I.-W.J., Dreiling J.L., Lee M.-J., Williams C.A., Oberst M.D., Dickson R.B., Lin C.-Y. (2004). Assembly of adherens junctions is required for sphingosine 1-phosphate-induced matriptase accumulation and activation at mammary epithelial cell-cell contacts. Am. J. Physiol. Physiol..

[31] Nagahashi M., Yamada A., Miyazaki H., Allegood J.C., Tsuchida J., Aoyagi T., Huang W.-C., Terracina K.P., Adams B.J., Rashid O.M. (2016). Interstitial Fluid Sphingosine-1-Phosphate in Murine Mammary Gland and Cancer and Human Breast Tissue and Cancer Determined by Novel Methods. J. Mammary Gland. Biol. Neoplasia.

[32] Kamburov A., Wierling C., Lehrach H., Herwig R. (2009). ConsensusPathDB-a database for integrating human functional interaction networks. Nucleic Acids Res..

[33] Kim S.E., Lee E.J., Chae M.K., Yoon J.S. (2016). The Role of Sphingosine-1-Phosphate in Adipogenesis of Graves’ Orbitopathy. Investig. Opthalmology Vis. Sci..

[34] Jun D.-J., Lee J.-H., Choi B.-H., Koh T.-K., Ha D.-C., Jeong M.-W., Kim K.-T. (2006). Sphingosine-1-Phosphate Modulates Both Lipolysis and Leptin Production in Differentiated Rat White Adipocytes. Endocrinology.

[35] Moon M.-H., Jeong J.-K., Park S. (2014). Activation of S1P2 receptor, a possible mechanism of inhibition of adipogenic differentiation by sphingosine 1-phosphate. Mol. Med. Rep..

[36] Shannon P., Markiel A., Ozier O., Baliga N.S., Wang J.T., Ramage D., Amin N., Schwikowski B., Ideker T. (2003). Cytoscape: A Software Environment for Integrated Models of Biomolecular Interaction Networks. Genome Res..

[37] Alzahrani A.S. (2019). PI3K/Akt/mTOR inhibitors in cancer: At the bench and bedside. Semin. Cancer Biol..

[38] Verret B., Cortes J., Bachelot T., Andre F., Arnedos M. (2019). Efficacy of PI3K inhibitors in advanced breast cancer. Ann. Oncol..

[39] Hoxhaj G., Manning B.D. (2019). The PI3K–AKT network at the interface of oncogenic signalling and cancer metabolism. Nat. Rev. Cancer.

[40] Crown S.B., Marze N., Antoniewicz M.R. (2015). Catabolism of Branched Chain Amino Acids Contributes Significantly to Synthesis of Odd-Chain and Even-Chain Fatty Acids in 3T3-L1 Adipocytes. PLoS ONE.

[41] Popovich D.G., Lee Y., Li L., Zhang W. (2011). Momordica charantia Seed Extract Reduces Pre-Adipocyte Viability, Affects Lactate Dehydrogenase Release, and Lipid Accumulation in 3T3-L1 Cells. J. Med. Food.

[42] Thul P.J., Lindskog C. (2018). The human protein atlas: A spatial map of the human proteome. Protein Sci..

[43] Lehr S., Hartwig S., Lamers D., Famulla S., Müller S., Hanisch F.-G., Cuvelier C., Ruige J., Eckardt K., Ouwens D.M. (2012). Identification and Validation of Novel Adipokines Released from Primary Human Adipocytes. Mol. Cell. Proteom..

[44] Bauer-Mehren A., I Furlong L., Sanz F. (2009). Pathway databases and tools for their exploitation: Benefits, current limitations and challenges. Mol. Syst. Biol..

[45] Chen L., Chu C., Lu J., Kong X., Huang T., Cai Y.-D. (2015). Gene Ontology and KEGG Pathway Enrichment Analysis of a Drug Target-Based Classification System. PLoS ONE.

[46] Fabregat A., Sidiropoulos K., Viteri G., Forner-Martinez O., Marin-Garcia P., Arnau V., D’Eustachio P., Stein L., Hermjakob H. (2017). Reactome pathway analysis: A high-performance in-memory approach. BMC Bioinform..

[47] Slenter D.N., Kutmon M., Hanspers K., Riutta A., Windsor J., Nunes N., Mélius J., Cirillo E., Coort S.L., Digles D. (2018). WikiPathways: A multifaceted pathway database bridging metabolomics to other omics research. Nucleic Acids Res..

